# Validation of the German Version of the P4 Suicidality Tool

**DOI:** 10.3390/jcm12155047

**Published:** 2023-07-31

**Authors:** Sabine Schluessel, Kathrin Halfter, Carolin Haas, Kurt Kroenke, Karoline Lukaschek, Jochen Gensichen

**Affiliations:** 1 Institute of General Practice and Family Medicine, University Hospital, LMU Munich, 80336 Munich, Germany; sabine.schluessel@med.uni-muenchen.de (S.S.); c.haas@med.uni-muenchen.de (C.H.); jochen.gensichen@med.uni-muenchen.de (J.G.); 2 Institute for Medical Information Processing, Biometry, and Epidemiology, Ludwig-Maximilians University, 81377 Munich, Germany; halfter@ibe.med.uni-muenchen.de; 3 Graduate Program “POKAL—Predictors and Outcomes in Primary Care Depression Care” (DFG-GrK 2621), 80336 Munich, Germany; 4 Department of Medicine, Indiana University School of Medicine, Indianapolis, IN 46202, USA; kkroenke@regenstrief.org; 5 Regenstrief Institute, Indianapolis, IN 46202, USA; 6 DZPG (German Center for Mental Health), 80336 Munich, Germany

**Keywords:** suicide prevention, suicidal ideation, suicidal behavior screener, psychometric evaluation, primary care

## Abstract

For general practitioners (GPs), it may be challenging to assess suicidal ideation (SI) in patients. Although promising instruments exist for the use in primary care, only a few have been validated in German. The objectives of this study were to examine the validity of the brief P4 screener for assessing SI in a cross-sectional study including outpatients. Inclusion criteria were a PHQ-9 score ≥ 10 or an affirmative answer to its SI item. Construct validity of the P4 was examined by comparison with the four-item Suicide Behaviors Questionnaire-Revised (SBQ-R), the PHQ-9 (convergent), and the positive mental health (PMH) scale (divergent). The study sample included 223 patients (mean age 47.61 ± 15 years; 61.9% women) from 20 primary care practices (104 patients) and 10 psychiatric/psychotherapeutic clinics (119 patients). The first three items of the P4 correlate positively with most of the four items of the reference standard SBQ-R (convergent validity); the fourth item of the P4 (preventive factors) correlates significantly with the PMH scale. The most common preventive factor (67%) is family or friends. The German P4 screener can be used to assess SI in outpatient care. It explores preventive or protective factors of suicide, which may support the GP’s decision on treatment. We recommend a further clinical interview for patients flagged by P4 assessment in order to more formally assess suicidal risk.

## 1. Introduction

Suicidal ideation (SI) is a broad term used to describe a range of thoughts, contemplations, wishes, and preoccupations with death and suicide [1,2,3]. People expressing SI are four times more likely to die by suicide than people not expressing SI [4]. Studies show that SI is present in up to 10% of primary care patients [5,6,7], especially in patients with psychiatric disorders [8].

General practitioners (GPs) play a central role in depression care and suicide prevention, due to the usually long-standing relationship of trust between GP and patient [9]. Studies show that on the one hand, patients actively seek contact with their GP in suicidal crises [10]. On the other hand, primary care patients rarely address SI on their own. Thus, it is a responsibility of, and challenge for, the GP to directly address this topic [4,8,11,12]. 

Assessing SI in vulnerable patients is an important step in suicide prevention [13]. According to the current German national guideline on the treatment of patients with depression [14], SI should be addressed directly and seriously by the GP. In order to assist GPs with this difficult task, it is imperative to provide instruments that can be efficiently integrated into everyday practice, are time-saving, and reliable to rule-out suicidal patients with high certainty [15,16,17]. Screening instruments have already been developed in the English-speaking world [16,18] including the following: P4 [19], Paykel Suicide Items (PSI) [20], Depressive Symptom Inventory Suicidality Subscale (DSI-SS) [21], or Suicide Behaviors Questionnaire-Revised (SBQ-R) [22]. In addition, there are other self-assessment and external assessment procedures, which are far more complex in their handling and evaluation and, for this reason, are too time-consuming for use in general practice [18]. P4, PSI, SBQ-R, and DSI-SS are relatively short and have already been used in studies and clinical practice for screening and early detection of SI. However, validated and published German translations only exist for DSI-SS [23] and SBQ-R [24]. The DSI-SS records the frequency and intensity of suicidal thoughts and impulses in the past two weeks with four items; suicidal behavior is not asked. The four items of the SBQ-R capture different facets of suicidal experience and behavior (lifetime suicidal thoughts, plans, and attempts; suicidal thoughts during the last 12 months; expression of suicidal intentions; probability of future suicidal acts) [18]. However, DSI-SS and SBQ-R are only of limited use in everyday clinical practice: neither suicidal intentions nor suicide attempts are recorded with the DSI-SS, and no current suicidal experience can be recorded with the SBQ-R. Neither of the two instruments ask for protective factors, but the scientific community explicitly recommends focusing on protective factors [25,26]. All this information is, however, important for the GP.

The P4 is a four-item tool assessing SI. The four letters “P” of the P4 questionnaire stand for **p**ast suicide attempt, suicide **p**lan, **p**robability of completing suicide, and **p**reventive factors. The original English version of the P4 was evaluated in two clinical trials [19] in which suicide screening was carried out at five time points: at study enrolment and 1, 3, 6, and 12 months later. Patients were classified into different risk groups by answering the four items of the P4 screener: minimal, lower, and higher suicidality risk. Overall, suicide assessment by the P4 was initiated at one or more time points by 17.6% (44 of 250) of the participants in the Stepped Care for Affective Disorders and Musculoskeletal Pain (SCAMP) trial and by 16.5% (51 of 309) of the participants in the Indiana Cancer Pain and Depression (INCPAD) trial. Of the patients in whom a suicide evaluation was initiated, the majority (29 of 44 in SCAMP and 27 of 51 in INCPAD) were classified as minimal risk by the algorithm. Only one (0.4%) of SCAMP participants and five (1.6%) of INCPAD participants were classified as higher risk. The decisive advantage of the P4 compared to other short suicide instruments is that it is assessing factors that could prevent the patient from suicide (“Is there anything that would prevent you from doing something to yourself”?). Knowledge of these protective personal factors is an important component for therapy and is considered a preventive factor in clinical practice. Additionally, the P4 can be integrated in clinic procedures to assess suicidality in vulnerable patients while minimizing the impact on the GP’s workflow. 

Due to its characteristics, the P4 seems to be a very suitable instrument for primary care. Additionally, existing short instruments validated in German (DSI-SS, SBQ-R) are not without flaws. Therefore, the aim of the study was to provide a validated German version of the P4 that can support GPs in particular to start a dialogue with suicidal patients including a preventive perspective. 

## 2. Material and Methods

### 2.1. Study Design and Setting

We performed a cross-sectional study in outpatient care (primary care practices and outpatient psychologist clinics) in Germany. Using the P4 and SBQ-R in a face-to-face interview, providers assessed their patients. Ethical approval came from the ethics committee of the Faculty of Medicine, Ludwig Maximilian University (LMU) (#19-467, 5 September 2019). All methods were carried out in accordance with relevant guidelines and regulations. Informed consent was obtained from all participants (participants under 18 years were not included in the study). 

### 2.2. Recruitment and Data Collection

We contacted via email or personal invitation a total of 1048 providers from various primary care and psychotherapeutic clinics to participate in the study. Additionally, we promoted the project within the Bavarian Research Network in General Medicine (BayFoNet) [27]. In total, 30 clinics were enrolled (20 primary care, 9 psychotherapeutic clinics, 1 psychiatric hospital). GPs needed to be trained in “basic psychosomatic care” (80 h of specialized training provided to those practicing general medicine, obligatory for GPs in Germany).

The provider invited patients with a F3 (affective disorders) and/or F4 (neurotic, stress, and somatoform disorders) diagnosis according to International Classification of Diseases 10th revision (ICD-10) to participate in the study. Patients (age ≥ 18) who answered the key question about SI positively (“Thoughts that you would be better off dead or of hurting yourself in some way”?) or scored > 9 points on the Patient Health Questionnaire nine-item depression scale (PHQ-9) were enrolled. With a score greater than 9 points, the presence of depression can be assumed [28]. Patients with dementia, psychotic diseases, or insufficient German language skills were excluded. An expense allowance of 20 Euro per included patient was paid to the providers. Data collection took place between September 2019 and February 2020.

### 2.3. Study Population

The study included 223 patients (mean age 47.6 ± 15.1 years; 61.9% women): 104 patients from 20 primary care practices and 119 patients from specialty settings (9 ambulant psychotherapeutic practices and 1 stationary ward). Patients’ characteristics are shown in Table 1. Main diagnosis was classified as F3 (affective disorders) and/or F4 (neurotic, stress, and somatoform disorders) according to the ICD-10. 

### 2.4. The P4 Questionnaire

**Figure 1:**
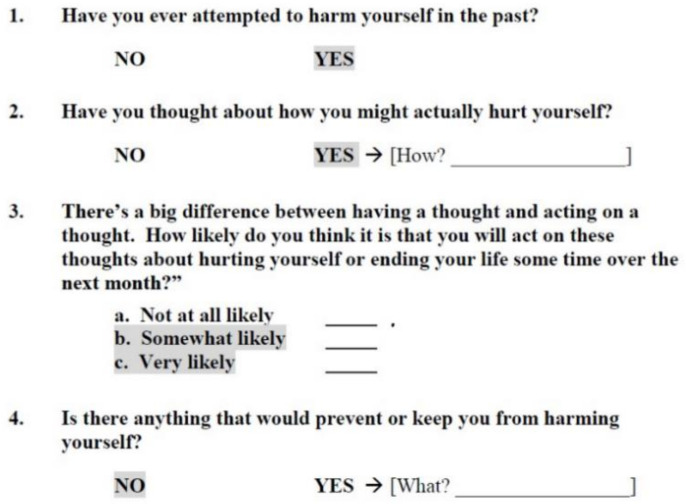
Original English version of the P4 according to Dube et al. (2014), Figure 1. [19]. The shaded items translate into risk categories as follows: minimal risk—no shaded items; lower risk—at least one item of items 1 and 2 is shaded, but no shaded responses to items 3 and 4; higher risk: at least one item is shaded.

The P4 (past suicide attempts, suicide plan, probability of completing suicide, and preventive factors) can be used to assess the clinical risk after the patient has expressed suicidal tendencies, e.g., either during a conversation, part of the PHQ-9, or other depression screener. The questions about past suicide attempts, suicide plan, and protective factors are initially dichotomous yes/no questions, with the option to provide detail in case of “yes” (Figure 1). The question about the probability of completing suicide provides three answers (not at all likely/somewhat likely/very likely). Patients then are classified into one of three risk categories depending on their answers: minimal risk, lower risk, and higher risk (see Figure 1).

### 2.5. The German Translation

**Figure 2:**
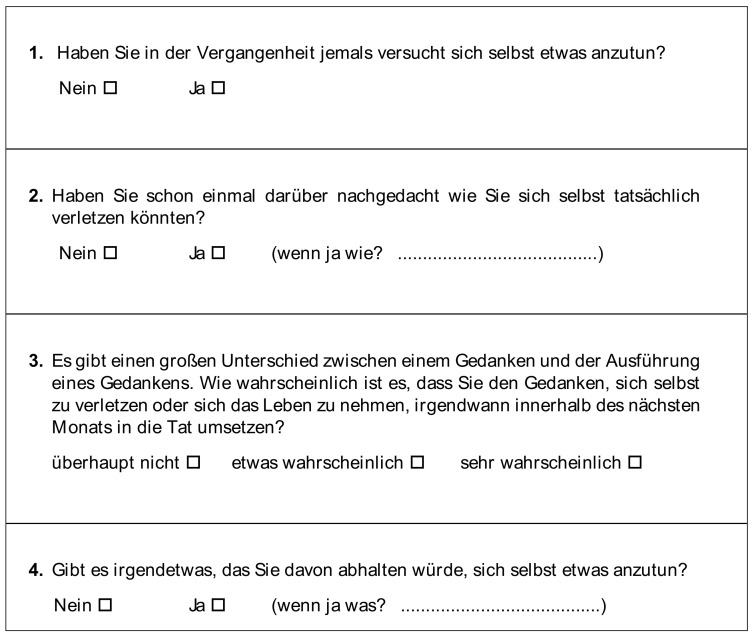
German version of the P4 as developed by the authors.

The German version of the P4 was obtained by using the established process of translation and adaptation of instruments of the World Health Organisation (WHO) [13]. The questionnaire was translated by a health professional and adapted by an expert committee of five physicians and two psychologists, then back-translated by a native English speaker (Figure 2). The final German version was preliminarily tested for comprehensibility at the University of Bochum (N = 300 students).

### 2.6. Other Instruments

SBQ-R. To assess convergent validity, we used the Suicide Behaviors Questionnaire-Revised (SBQ-R). The SBQ-R is a questionnaire for the assessment of suicidal behavior. Similar to the P4, it comprises only four questions that explore lifetime SI or suicide attempt(s), frequency of SI over the past 12 months, threat of suicide attempt, and self-reported likelihood of suicide behavior in the future. The sum score ranges from 3 to 18 with a cut-off score ≥ 7 for adults of general population. The German version has demonstrated sufficient psychometric properties (internal consistency Cronbach’s α = 0.72) [24].

PHQ-9. The Patient Health Questionnaire nine-item depression scale (PHQ-9) is one of the most reliable (Cronbach’s alpha = 0.89) and widely used depression measures in clinical practice and research; (Cronbach’s alpha = 0.89) [29,30]. Each item asks how often the respondent has been bothered by a particular criterion symptom in the past 2 weeks; item scores from 0 to 3 for the four response options: (0) “Not at all”, (1) “Several days”, (2) “More than half the days”, and (3) “Nearly every day”. A sum score ranging from 0 to 27 points quantifies severity of depression.

PMH. Resilience factors play a decisive role in suicide prevention. This also includes the subjective evaluation of one’s own well-being, which is assessed by the positive mental health (PMH) scale [31]. The construct of positive mental health has a moderating effect on the extent to which depression occurs with or without suicidality, and whether suicidal ideation turns into suicidal behavior [25,26]. The PMH has good reliability (Cronbach’s alpha = 0.93) and consists of nine Likert-type items. Each item has four response categories with scores from 0 to 3: (0) do not agree, (1) disagree, (2) tend to agree, and (3) agree. Higher scores represent higher levels of patient “well-being” (in total a sum score from 0 to 27 points).

The following demographic data were also collected: age, sex, family status, number of children, and psychiatric pre-existing conditions.

### 2.7. Data Analysis

The evaluation of the measuring instrument followed the principles of Terwee et al. [32]. These principles provide an evaluation framework for questionnaires for recording the health status according to uniform quality criteria. 

For statistical analysis, the answers of the P4 were scored. For the first three items, the answers “no”/“not all likely” were scored with “0”, indicating minimal suicide risk. All other answers were scored with “1”, indicating risk. The last item was reverse-scored as it is a preventative question. Therefore, the answer format “no” indicates risk and, therefore, received the score “1”. 

The construct validity of P4 was measured in accordance with the Suicide Behavior Questionnaire-Revised (SBQ-R) [22,24] that also measures the construct of SI (convergent validity). Construct validity was furthermore assessed by correlations of P4 items and sum score with the PHQ-9 and PMH scores; we expected moderately positive correlations with the PHQ-9 (convergent) and low correlations with the PMH (divergent validity). The agreement between the risk groupings of P4 and SBQ-R was measured using Cohen’s kappa coefficient (K) [33]. Associations of the P4 with patient demographic factors were also examined. An alpha level of *p* < 0.05 was used for tests of statistical significance. Subgroup analyses (primary care and specialized setting) were conducted in the same way. Statistical analysis was performed using IBM SPSS 25 for Windows (Chicago, IL, USA).

Finally, responses to the two open-ended P4 questions on suicidal plans and protective factors were categorized in the same way as by Dube et al. in the original P4 study [19]. 

## 3. Results

Table 2 shows the mean values for depression (PHQ-9), P4, suicidal behavior (SBQ-R), and positive mental health (PMH). As to be expected, depression levels are rather high, and positive mental health rather low. 

### 3.1. P4 Results

Table 3 shows the distribution of participants regarding each of the four P4 items to no risk/risk categories, as well as a total risk estimation (minimal risk vs. lower or high risk):

One third of the study population (30.5%, n = 68) are classified as minimal suicide risk, 58.7% (n = 120) as lower risk, and 15.7% (n = 35) as higher risk. It is notable that >90% (n = 202) indicate protective factors. 

The sum score, as well as the first three items of the P4 correlate positively (*p*-value < 0.01) with most of the four items and the sum score of the reference standard SBQ-R (convergent validity). All these correlations are moderate to high. No connection is found for the fourth item of the P4 (preventive factors), not correlating to any item of the SBQ-R. However, it correlates significantly with the PMH scale (see Table 3).

Apart from the correlations to the SBQ-R, the P4 risk categories show moderate correlations to the PHQ-9 sum score and the last question of the PHQ-9 (“Thoughts that you would be better off dead or of hurting yourself in some way ”; convergent validity, see Table 3). Conversely, the P4 items and score correlate poorly with the PMH, supporting the expected divergent validity from the positive mental health construct measured by the PMH. P4 risk categories are not associated with age, family status, or parenthood. The agreement between the risk groupings of P4 and SBQ-R is moderate, with a Cohen’s kappa K of 0.44.

### 3.2. Suicide Plan and Preventive Factors

Answers to open questions about preventive factors or means of suicide are summarized in categories (Table 4). The most common intended means of suicide are medication overdose (28.8%, n = 47) followed by intentional vehicular accident (17.8%, n = 29), and cutting oneself (17.8%, n = 29). Two-thirds of respondents who report preventive factors state family or friends (67.4%, n = 147), followed by future hopes (15.1%, n = 33).

### 3.3. Subgroup Analyses

The subgroup analyses of primary care and specialized setting show similar results (▶ Supplementary Materials). 

## 4. Discussion

Our study shows good convergent validity of the P4 screener with another brief suicidality screener—the SBQ-R. Furthermore, divergent validity is demonstrated with positive mental health (PMH). Contrary to our expectations, the severity of depression and the positive answers to the ninth PHQ-9 question do not differ in the two settings (20 primary care practices and specialized settings (10 psychotherapeutic practices and 1 stationary ward)). Thus, the results of the P4 in both settings are very similar and the analysis performed is combined. The qualitative results regarding protective factors are consistent with the findings of the original English questionnaire of Dube et al. [19] and, therefore, show that these categories, despite linguistic and cultural differences, may remain stable. It is notable that in the present study, only a minority indicate “hanging” as potential means of suicide, whereas according to the Statistische Bundesamt (DESTATIS, Federal Statistical Office) “hanging, strangulation or suffocation” was the most frequently chosen suicide method in Germany for both women and men in 2021; almost half of all men who committed suicide died this way (48.4%). Among women, it was 30.8% who chose this way of killing themselves (https://www.destatis.de/DE/Themen/Gesellschaft-Umwelt/Gesundheit/Todesursachen/suizid.html; accessed on 1 May 2023).

### 4.1. Suicidal Risk Stratification

Regarding the suicidal risk stratification, Dube et al. [19] showed that the majority of those who triggered a suicide assessment were classified as minimal risk (=risk category 1), indicating that patients had no past suicide attempt or current plan. In our study, most patients (over 50%) are classified as lower risk (=risk category 2). Lower risk category indicates a past suicide attempt or a current suicide plan, but considering the probability of hurting oneself as “not at all likely” and reporting protective factors. One reason for the higher risk score in our study might be that only patients with an elevated depression score (PHQ-9 > 9) or patients who endorsed the suicidal ideation item of the PHQ-9 were enrolled. In addition, more than half of the study patients were enrolled from mental health specialty practices. 

In the case of suicidal patients, GPs are often faced with the difficult question of whether they can still bear the responsibility for outpatient treatment. If the risk of suicide is rather low in the sense of temporarily occurring passive suicidal thoughts without plans, GPs can continue treatment of the patient on an outpatient basis. The English and the German study both show that a small proportion of patients are classified as high risk (=risk category 3). High risk is defined as a self-assessed probability of self-harm as “somewhat likely” or “very likely” or an absence of preventive factors. For patients with high risk the GP has several options, depending on the patients’ compliance: further steps include a more detailed exploration, closer monitoring, and, if needed, admission to a psychiatric hospital—perhaps against the patient’s will, although this could strain the relationship of trust that is important for the treatment of the suicidal patient. The current study situation regarding suicide prevention strategies in primary care is insufficient [34]. As a first step, a new German national guideline on suicide prevention is currently being developed [35], which will classify the evidence of suicide prevention measures in outpatient and inpatient settings.

In summary, we were able to show that the P4 is measuring SI in primary care patients with similar properties to the English original version. The optimal use of the P4 may be its integration as a guiding tool in the doctor–patient consultation, rather than as single screening instrument. A possible application scenario could be as follows: the PHQ-9 is a well-established instrument to assess depressive disorders in clinical practice. However, suicidality is only approached in one item (“Thoughts that you would be better off dead or of hurting yourself in some way”—not at all, several days, more than half the days, nearly every day) [29]. In case a patient scores on this item, GPs could use the P4 to initiate a conversation and further explore the patient’s SI. Studies have shown that patients are generally comfortable with being asked questions about SI by their GPs [36,37].

We are aware that sensitivity and specificity of screening instruments are always limited and lead to false negative or false positive results. One has to keep in mind that suicide *prediction* in individuals is near impossible [38,39]. Nevertheless, a feasible instrument can still support the GP’s decision and provide conversation guidance how to approach this sensitive topic, and, thus, can contribute to suicide *prevention.*

### 4.2. Strengths, Limitations, and Future Development

Our study has several strengths. The sample was reasonably large and included patients from both primary care and mental health specialty settings with the same inclusion criteria. Notably, subgroup analyses show similar results in both settings. Unlike the original study, we examined both construct validity, which, in turn, proved to be good. Similar to the original study, we were able to examine risk stratification as well as patient-reported means of suicide and protective factors. 

Several study limitations should be acknowledged. First, a more detailed psychiatric evaluation of suicide risk to compare with the P4 risk stratification was not implementable in an outpatient setting. Second, a repeated administration of the P4 was not feasible, therefore, not allowing assessment of test–retest reliability. Finally, we do not report Cronbach’s alpha coefficient for internal consistency: Due to the low item number of the P4 and its categorical scale, it is not a sensible measure. Furthermore, it is possible that only providers especially interested in the topic and/or research in general participated, which might have led to bias. 

Building on the ideas of the P4 to offer a short questionnaire for GPs that also includes protective aspects, we are currently developing and validating from scratch a new short questionnaire with the aim to have a psychometrically optimized instrument for suicide prevention in primary care (SuPr-X [40]). 

## 5. Conclusions

The German P4 screener is a suitable tool to assess suicidality within a reasonable timeframe in primary care. It facilitates an initial approach to the patient’s suicidal ideation and behavior. Most importantly, it explores protective factors of suicide, which may support the GP’s decision on treatment. Additionally, through its clear structure, the P4 can provide GPs with more confidence in counseling vulnerable and suicidal patients.

Although the P4 can provide an initial risk stratification, we recommend a more detailed clinical interview for those who screen positive in order to more formally assess suicidal risk. 

## Figures and Tables

**Table 1:** Description of the study population (N = 223).

	Total (n = 222) *		Women (n = 138)		Men (n = 84)	
	Mean	SD	Mean	SD	Mean	SD
Age (n = 220)	47.61	15.1	47.88	15.70	47.17	14.15
	%	**N**	%	**N**	%	**N**
Setting						
Primary care	46.7	104	48.6	67	42.9	36
Specialized care	53.4	119	51.5	71	57.1	48
Main diagnosis						
F3	46.9	104	47.1	65	46.4	39
F4	7.2	16	6.5	9	8.3	7
Both F3 and F4	27.0	60	28.3	39	25.0	21
No F diagnosis	15.8	35	14.5	20	17.9	15
Other F diagnosis	3.2	7	3.6	5	2.4	2
Marital status						
Married/relationship	40.5	90	38.4	53	44.1	37
Separated/divorced/widowed	21.6	48	27.5	38	11.8	10
Single	37.8	84	34.1	47	44.1	37
Parenthood						
Yes	53.2	118	57.2	79	46.4	39
No	46.9	104	42.8	59	53.6	45

* Three participants with missing data on socio-demographic information (n = 1) and age (n = 2) Abbreviations: PHQ9: Patient Health Questionnaire. SBQ-R: Suicide Behavior Questionnaire. PMH: positive mental health. F3 = affective disorders according to International Classification of Diseases 10th revision (ICD-10). F4 = neurotic, stress, and somatoform disorders (according to ICD-10).

**Table 2:** Scores of the study population (N = 223).

	Total (n = 223)		Women (n = 139)		Men (n = 84)	
	Mean	SD	Mean	SD	Mean	SD
Scale scores (range)						
PHQ-9 depression (0–27)	15.00	4.36	15.10	4.45	14.90	4.21
SBQ-R (3–18)	7.38	4.16	7.36	4.13	7.37	4.23
PMH (0–27)	8.18	7.00	8.26	7.23	8.06	6.68

Abbreviations: PHQ9: Patient Health Questionnaire. SBQ-R: Suicide Behavior Questionnaire. PMH: positive mental health.

**Table 3:** Prevalence of P4 risk categories and correlation with other scale items and scores.

	Total (n = 223) *		Women (n = 139)		Men (n = 84)		Intercorrelations (Convergent Validity) *							
	**No Risk**	**Risk**	**No Risk**	**Risk**	**No Risk**	**Risk**	**SBQ-R_1 (Lifetime SI/Attempts)**	**SBQ-R_2** **(12 Month SI)**	**SBQ-R_3 (Threatened Suicide)**	**SBQ-R_4** **(Suicide Likelihood)**	**SBQ-R** **_sum**	**PMH** **_sum**	**PHQ9** **_sum**	**PHQ9_09**
**P4_1** **(past attempt)**	159 (71.3%)	64 (28.7%)	95 (68.8%)	43 (31.2%)	64 (78.2%)	20 (23.8%)	**0.696**	**0.292**	**0.282**	**0.267**	**0.465**	0.016	0.118	**0.238**
**P4_2** **(suicide plan)**	84 (37.7%)	139 (62.3%)	46 (33.3%)	92 (66.7%)	38 (45.2%)	46 (54.8%)	**0.588**	**0.556**	**0.510**	**0.205**	**0.580**	−0.015	**0.130**	**0.220**
**P4_3** **(suicide likelihood)**	200 (89.7%)	23 (10.3%)	123 (89.1%)	15 (10.9%)	76 (90.5%)	8 (9.5%)	**0.178**	**0.248**	**0.193**	**0.355**	**0.329**	−0.108	**0.240**	**0.364**
**P4_4** **(preventive factors)**	202 (90.6%)	21 (9.4%)	127 (92.0%)	11 (8.0%)	74 (88.1%)	10 (11.9%)	0.024	0.047	0.043	0.051	0.550	**0.191**	0.053	0.121
**P4_risk**	minimal: 68 (30.5%)	lower: 120 (53.8%) higher: 35 (15.7%)	minimal: 36 (26.1%)	lower: 81 (58.7%) higher: 21 (15.2%)	minimal: 32 (38.1%)	lower: 38 (45.2%) higher: 14 (16.7%)	**0.683**	**0.512**	**0.464**	**0.365**	**0.634**	−0.095	**0.219**	**0.383**

* 1ID is missing gender information. All **bolded correlation coefficients** are significant at *p* < 0.01.

**Table 4:** Explorative analysis of suicide plan and preventive factors.

Suicide Plan	N	%
Medication overdose	47	28.8
Intentional vehicular accident	29	17.8
Cutting oneself	29	17.8
Others	21	12.9
Falls	14	8.6
No answer	12	7.4
Hanging	9	5.5
Using a gun	2	1.2
**Total**	163	100
**Preventive Factors**	N	%
Family	147	67.4
Future hope	33	15.1
Faith	12	5.5
Fear of failing	8	3.7
Others	18	8.3
**Total**	218	100

## Data Availability

Data cannot be shared publicly because of German data protection laws. However, data are available upon reasonable request. Data requests may be directed to “Stiftung Allgemeinmedizin—The Primary Health care Foundation” (www.stiftung-allgemeinmedizin.de). Mail: office@stiftung-allgemeinmedizin.de.

## Supplementary Materials

The following supporting information can be downloaded at: https://www.mdpi.com/article/10.3390/jcm12155047/s1, Table S1: ST1. Prevalence of P4 Risk Categories and Correlation with Other Scale Items and Scores (subgroups). Table S2: Qualitative evaluation (Subgroups) of Suicidal Ideation Plans and Preventive Factors.

## Abbreviations

GP	General practitioner
PHQ-9	Patient Health Questionnaire-9
PMH	Positive mental health-scale
SBQ-R	Suicide Behaviors Questionnaire-Revised
SI	Suicide ideations
