# Supplementary material for: Validation of the German Version of the P4 Suicidality Tool
Source: J Clin Med. 2023 Jul 31;12(15):5047. doi: 10.3390/jcm12155047 (PMC10420186; doi:10.3390/jcm12155047)
Supplement: Supplementary file 1 [file jcm-12-05047-s001.zip › jcm-2408958-supplementary.pdf]

Supplementary Part (Subgroups):

ST1. Prevalence of P4 Risk Categories and Correlation with Other Scale Items and Scores (subgroups)

Specialized care

	Total (n=119)		Women (n=71)		Men (n=48)		Intercorrelations (convergent validity) **							
	no risk	risk	no risk	risk	no risk	risk	SBQ-R_1 (lifetime SI/attempts)	SBQ-R_2 (12-month SI)	SBQ-R_3 (threatened suicide)	SBQ-R_4 (suicide likelihood)	SBQ-R _sum	PMH _sum	PHQ9 _sum	PHQ9_ 09
P4_1 (past attempt)	84 (70.6%)	35 (29.4%)	46 (64.8%)	25 (35.2%)	38 (79.2%)	10 (20.8%)	.756**	.284**	.316**	.318**	.371**	.006	.142	.284**
P4_2 (suicide plan)	43 (36.1%)	76 (63.9%)	22 (31.0%)	49 (69%)	21 (43.8%)	27(56.3%)	.521**	.603**	.504**	.319**	.609**	.076	.060	.246**
P4_3 (suicide likelihood)	102 (85.7%)	17 (14.3%)	59 (83.1%)	12 (16.9%)	43 (89.6%)	5 (10.4%)	.089	.201*	.203*	.374**	.133	-.131	.204*	.310**
P4_4 (preventive factors)	105 (88.2%)	14 (11.8%)	63 (88.7%)	8 (11.3%)	42 (87.5%)	6 (12.5%)	.019	.064	.086	.112	.082	-.205*	.030	.132
P4_risk (minimal, lower, higher)	34 (28.6%)	lower: 61 (51.3%) higher: 24 (20.2%)	17 (23.9%)	lower: 39 (54.9%) higher: 15 (21.2%)	17 (35.4%)	lower: 22 (45.8%) higher: 9 (18.8%)	.335**	.405**	.345**	.420**	.448**	-.121	.124	.365**

** correlation is significant at P < .01 level, *corelation is significant at p< .05

Primary care

	Total (n=104)		Women (n=68)		Men (n=36)		Intercorrelations (convergent validity) *							
	no risk	risk	no risk	risk	no risk	risk	SBQ-R_1 (lifetime SI/attempts)	SBQ-R_2 (12-month SI)	SBQ-R_3 (threatened suicide)	SBQ-R_4 (suicide likelihood)	SBQ-R _sum	PMH _sum	PHQ9 _sum	PHQ9_ 09
P4_1 (past attempt)	76 (73.1%)	28 (26.9%)	50 (73.5%)	18 (26.5%)	26 (72.2%)	10 (27.8%)	.725**	.291**	.295**	.228*	.309**	.06	.07	.18
P4_2 (suicide plan)	41 (39.4%)	63 (60.6%)	24 (35.5%)	44 (64.7%)	17 (47.2%)	19 (52.8%)	.563**	.505**	.448**	.185	.388**	-.10	.212*	.216*
P4_3 (suicide likelihood)	98 (94.2%)	6 (5.7%)	65 (95.6%)	3 (3.4%)	33 (91.7%)	3 (8.4%)	.252**	.293**	.231*	.400**	.196*	-.05	.203*	.422**
P4_4 (preventive factors)	96 (93.2%)	7 (6.8%)	64 (95.5%)	3 (4.5%)	32 (88.9%)	4 (11.1%)	.049	.010	.061	-.048	-.003	-.053	.053	.129
P4_risk (minimal, lower, higher)	34 (33.0%)	lower: 58 (56.3%) higher: 11 (10.7%)	19 (28.4%)	lower: 42 (62.7%) higher: 6 (9%)	15 (341.7%)	lower: 16 (44.4%) higher: 5 (13.9%)	.600**	.492**	.408**	.229*	.354**	-.10	.283**	.358**

** correlation is significant at P < .01 level, *corelation is significant at p< .05

P4_1: Have you ever attempted to harm yourself in the past? (no/yes)

P4_2: Have you thought abought how you might actually hurt yourself? (no/yes)

P4_3: There's a big difference between having a thought and acting on a thought. How likely do you think it is that you will act on these thoughts about hurting yourself or ending your life some time over the next month? (not at all likely/somewhat likely/ very likely)

P4_4: Is there anything that would prevent or keep you from harming yourself? (no/yes)

ST2. Qualitative evaluation (Subgroups) of Suicidal Ideation Plans and Preventive Factors

Specialized setting

**Suicidal Ideations Plans**	**N**	**%**
medication overdose	27	29.7
cutting oneself	18	19.8
intentional vehicular accident	15	16.5
others	13	14.3
hanging	6	6.6
falls	6	6.6
no answer	5	5.5
using a gun	1	1.1
**Total**	**91**	**100**

Primary care

**Suicidal Ideations Plans**	**N**	**%**
medication overdose	20	27.8
intentional vehicular accident	14	19.4
cutting oneself	11	15.3
others	8	11.1
falls	8	11.1
no answer	7	9.7
hanging	3	4.2
using a gun	1	1.4
**Total**	**72**	**100**

Specialized setting

**Preventive Factors**	**N**	**%**
family	78	66.1
future hope	20	16.9
faith	6	5.1
fear of failiing	4	3.4
others	10	8.5
Total	**118**	**100**

Primary care

**Preventive Factors**	**N**	**%**
family	69	69.0
future hope	13	13.0
faith	6	6.0
fear of failiing	4	4.0
others	8	8.0
Total	**100**	**100**
